# Frequencies of poor metabolizer alleles of 12 pharmacogenomic actionable genes in Punjabi Sikhs of Indian Origin

**DOI:** 10.1038/s41598-018-33981-z

**Published:** 2018-10-24

**Authors:** Dharambir K. Sanghera, Cynthia Bejar, Bishwa Sapkota, Gurpreet S. Wander, Sarju Ralhan

**Affiliations:** 1 Department of Pediatrics, College of Medicine, University of Oklahoma Health Sciences Center, Oklahoma City, Oklahoma USA; 2 Department of Pharmaceutical Sciences, University of Oklahoma Health Sciences Center, Oklahoma City, OK USA; 3 Oklahoma Center for Neuroscience, University of Oklahoma Health Sciences Center, Oklahoma City, OK USA; 4 Harold Hamm Diabetes Center, University of Oklahoma Health Sciences Center, Oklahoma City, OK USA; 5 Hero DMC Heart Institute, Ludhiana, Punjab India

## Abstract

Diversity in drug response is attributed to both genetic and non-genetic factors. However, there is paucity of pharmacogenetics information across ethnically and genetically diverse populations of India. Here, we have analyzed 21 SNPs from 12 pharmacogenomics genes in Punjabi Sikhs of Indian origin (N = 1,616), as part of the Sikh Diabetes Study (SDS). We compared the allele frequency of poor metabolism (PM) phenotype among Sikhs across other major global populations from the Exome Aggregation Consortium and 1000 Genomes. The PM phenotype of CYP1A2*1 F for slow metabolism of caffeine and carcinogens was significantly higher in Indians (SDS 42%, GIH [Gujarati] 51%, SAS [Pakistani] 45%) compared to Europeans 29% (p_genotype_ = 5.3E-05). Similarly, South Asians had a significantly higher frequency of CYP2C9*3 (12% SDS, 13% GIH, 11% SAS) vs. 7% in Europeans (p_genotype_ = <1.0E-05) and ‘T’ allele of *CYP4F2* (36%) SDS, (43%) GIH, 40% (SAS) vs. (29%) in Europeans (p_genotype_ = <1.0E-05); both associated with a higher risk of bleeding with warfarin. All South Asians –the Sikhs (0.36), GIH (0.34), and SAS (0.36) had a higher frequency of the NAT2*6 allele (linked with slow acetylation of isoniazid) compared to Europeans (0.29). Additionally, the prevalence of the low activity ‘C’ allele of *MTHFR* (rs1801131) was highest in Sikhs compared to all other ethnic groups [SDS (44%), GIH (39%), SAS (42%) and European (32%) (p_genotype_ = <1.0E-05)]. SNPs in *MTHFR* affect metabolism of statins, 5-fluorouracil and methotrexate-based cancer drugs. These findings underscore the need for evaluation of other endogamous ethnic groups of India and beyond for establishing a global benchmark for pre-emptive genotyping in drug metabolizing genes before beginning therapeutic intervention.

## Introduction

Adverse drug reactions (ADRs) are the fourth leading cause of death that lead to over 130,000 deaths each year in the United States alone^1^. Recent advances in pharmacogenomics have allowed us to determine individual’s response to drugs, and to predict the best possible treatment option for that individual based on his/her genetic profile.

Diversity in drug response for affecting drug absorption, distribution, metabolism, and elimination (ADME) is well documented at the individual and population level, and is attributed to both genetic and non-genetic factors^2^. Several earlier studies have reported variation in potentially important pharmacogenomics actionable genes - predominantly from cytochrome P450 families, to be influencing variation in inter-individual response to commonly used drugs^3–7^. Importantly, the current need of personalized medicine is to employ the knowledge of human genetics and pharmacogenetics to understand how an individual can benefit from specific drugs worldwide. Thus far, a vast majority of genetic and exome-wide sequencing studies in the US to identify rare functional variants in pharmacogenomics genes and other chronic diseases, have been largely performed in American individuals of European or African descent^8^. However, there is a paucity of pharmacogenetics information of these genes across ethno-genetically diverse populations in the world, particularly from South Asia. For instance, in India alone, there are more than 5,000 anthropological groups with many small endogenous communities. Many of these exhibit large differences in the allele-frequencies from their neighbors reflecting strong founder events^9^. Indeed, in India only, a high level of population substructure has been reported by a number of genetic studies^10–13^. Thus, this large variation in allele frequencies among Indian sub-ethnic groups is medically significant from the personalized medicine standpoint. However, very limited information is currently available on these populations, despite the fact that there is a growing epidemic of cardiovascular and metabolic diseases in the South Asian sub-continent^14,15^.

Here, in this study, we have analyzed 21 SNPs from 12 pharmacogenomic actionable genes involved in ADMEs listed in the Pharmacogenomics Knowledge Base (PharmGKB: http://www.pharmgkb.org) in a population of Punjabi Sikhs from the Northern part of India, and compared the genotype frequency distribution associated with the poor metabolism (PM) phenotype across five major global populations including South Asian (GIH [Gujarati Indian from Hap Map] and SAS [Pakistan]), European (non-Finnish Europeans NFE), East Asians (EAS), African (AFR), Latino (AMR), and others (OTH) using data from the Exome Aggregation Consortium (ExAC).

## Materials and Methods

### Study subjects

All study participants were part of the Asian Indian Diabetic Heart Study/Sikh Diabetes Study (AIDHS/SDS)^16,17^ recruited from the northern parts of India. Clinical characteristics of the study subjects stratified by metabolic syndrome (MS) are presented in Supplementary Table 1. Demographic characteristics including anthropometric measurements, physical activity, smoking, alcohol consumption and diet of study subjects are described elsewhere^16,18,19^. Briefly, a diagnosis of MS was established based on the recommended clinical criteria of ATP III^20^. An individual having at least 3 of the 5 recommended clinical traits, for instance abdominal obesity given as waist circumference (>102 cm in men and >88 cm in women), triglyceride (≥150 mg/dL), high density lipoprotein cholesterol (HDL) (<40 mg/dL in men and <50 mg/dL in women), blood pressure (systolic/diastolic: ≥130/≥85 mmHg), and fasting blood glucose (≥110 mg/dL), was classified as having the MS. The individuals without these risk factors were used as controls. Both waist and hip circumferences were measured with a tape measure at the abdomen and at the hip. Waist-to-hip ratio (WHR) was derived as waist measurement divided by hip measurement. Body Mass Index (BMI) was calculated as (weight (kg)/height (meter)^2^). Blood pressure was measured twice after a five minute seated rest period with the participant’s feet flat on the floor. All blood samples were obtained at the baseline visit for biochemical measurement including lipid profile and DNA extraction. All participants provided informed consent following procedures approved by Institutional Review Boards (IRBs). All methods were performed in accordance with the relevant guidelines and regulations. All AIDHS/SDS protocols and consent documents were reviewed and approved by the University of Oklahoma Health Sciences Center’s IRB as well as the Human Subject Protection (Ethics) committees at the participating hospitals and institutes in India as described earlier^21^.

### Variant selection and genotyping

A total of 21 variants from 12 pharmacogenomics actionable genes were selected using the PharmGKB database (https://www.pharmgkb.org/), and analyzed in 1,616 individuals (864 MS cases and 752 controls). Genomic DNA was extracted from buffy coats or whole blood using QIAamp kits (Qiagen, Chatsworth, CA). Samples were genotyped using the Human 660 W Quad BeadChip panel (Illumina, Valencia, CA, USA), and stringent SNPs and sample quality controls were followed as part of the genome-wide association study (GWAS) to obtain the high quality variants as described earlier^22,23^. The genotype frequency distribution of the Sikh population was then compared with five major HapMap populations: South Asian (GIH (n = 83) and SAS (n = 8356), European (NFE) (n = 33,370), East Asians (EAS) (n = 4,327), African Americans (AFR) (n = 5,203), Latino (AMR) (n = 5,789), and others (OTH) (n = 454) using data from the ExAC (http://exac.broadinstitute.org). To confirm the absence or presence of some very rare variants associated with PM phenotypes (not available in ExAC), we have also used data from the 1000 Genomes Phase III (http://www.internationalgenome.org/phase-3-structural-variant-dataset/) or from the HapMap project (https://www.ncbi.nlm.nih.gov/probe/docs/projhapmap/).

### Statistical analysis

Phenotype variables for all quantitative traits with skewed distributions were normalized by log-transformation before statistical comparisons. Transformed variables (waist measurement, BMI, blood pressure, triglyceride, cholesterol, and blood glucose) were retransformed into the original measurement scale for presentation. The genotype distributions for all studied SNPs were in Hardy-Weinberg equilibrium in controls. Genotype frequency distributions of the healthy controls (individuals free from MS, N = 752) were compared with the ExAC or HapMap populations either by Chi-square test or Fisher’s exact test. A p-value < 0.0018 was considered statistically significant after Bonferroni correction [0.05/ (28)]. All the statistical analysis was performed using SPSS for windows statistical package (version 19.0) (SPSS Inc., Chicago, USA).

## Results

Clinical details of the study population (stratified by MS) are presented in Supplementary Table 1. The description of actionable genes, very important pharmacogenomics (VIP) variants, genomic position, and comparison of frequency distributions are presented in Tables 1 and 2. The details on the associated drug(s), clinical effect related to drugs, and the influence of genomic variation on drug response are presented in Table 3.

**Table 1:** Allele Frequency Distribution of VIP Gene Variants in Punjabi Sikhs and Other Major Global Populations from ExAC.

Marker	Gene	Chr	Position	Minor/Test Allele	Major Allele	MAF in MS Cases (n = 864)	Freq in South Asian			European	East Asian	African	Latino	Other
							MAF in MS Controls (n = 752)	GIH (n = 83)	SAS (n = 8256)	NFE (n = 33370)	EAS (n = 4327)	AFR (n = 5203)	AMR (n = 5789)	OTH (n = 454)
rs1801131	*MTHFR*	1	11777063	C	A	0.43	0.44	0.39^‡^	0.42	0.32	0.21	0.16	0.16	0.30
rs1801133	*MTHFR*	1	11778965	T	C	0.18	0.20	0.16^‡^	0.14	0.35	0.31	0.11	0.51	0.31
rs1801159	*DPYD*	1	97753983	C	T	0.10	0.09	0.10^‡^	0.10	0.20	0.25	0.16	0.31	0.18
rs1801265	*DPYD*	1	98121473	G	A	0.25	0.25	0.30^‡^	0.25	0.22	0.07	0.42	0.21	0.23
rs4124874	*UGT1A1*	2	234330398	T	G	0.44	0.41	0.39^‡^	0.35^†^	0.56^†^	0.70^†^	0.11^†^	0.49^†^	N/A
rs887829	*UGT1A1*	2	234333309	T	C	0.40	0.42	0.43^‡^	0.44^†^	0.31^†^	0.12^†^	0.53^†^	0.32^†^	N/A
rs1045642	*ABCB1*	7	86976581	G	A	0.35	0.39	0.40^‡^	0.40	0.47	0.62	0.80	0.55	0.49
rs2032583	*ABCB1*	7	86998497	G	A	0.14	0.15	0.18^‡^	0.15	0.12	0.05	0.18	0.10	0.12
rs1799930	NAT2*6	8	18302383	A	G	0.35	0.36	0.34^‡^	0.36	0.29	0.25	0.26	0.14	0.26
rs1799931	NAT2*7	8	18302650	A	G	0.06	0.06	0.07^‡^	0.08	0.03	0.15	0.03	0.15	0.06
rs4244285	CYP2C19*2	10	96541616	A	G	0.32	0.32	0.33^‡^	0.34	0.15	0.31	0.18	0.10	0.17
rs4986893	CYP2C19*3	10	96530400	A	G	0.004	0.005	0.005^†^	0.004	0.000	0.07	0.000	0.000	0.001
rs1799853	CYP2C9*2	10	96692037	T	C	0.06	0.06	0.05^‡^	0.05	0.13	3.5E-04	0.02	0.07	0.09
rs1057910	CYP2C9*3	10	96731043	C	A	0.10	0.12	0.13^‡^	0.11	0.07	0.034	0.013	0.036	0.066
rs28371685	CYP2C9*11	10	96730971	T	C	0.00	0.001	0.006^‡^	0.002	0.002	1.2E-04	0.021	0.002	0.003
rs1695	*GSTP1*	11	67109265	G	A	0.33	0.27	0.33^‡^	0.29	0.32	0.18	0.44	0.54	0.30
rs2306283	*SLCO1B1*	12	21221005	G	A	0.40	0.40	0.55^‡^	0.48	0.41	0.75	0.77	0.43	0.50
rs4149056	*SLCO1B1*	12	21222816	C	T	0.04	0.04	0.02^‡^	0.05	0.16	0.13	0.03	0.11	0.17
rs762551	CYP1A2*1 F	15	72828970	C	A	0.44	0.42	0.51^‡^	0.45^†^	0.29^†^	0.38^†^	0.52^†^	0.26^†^	N/A
rs9923231	*VKORC1*	16	31015190	T	C	0.21	0.20	0.19^‡^	0.20^†^	0.40^†^	0.95^†^	0.06^†^	0.46^†^	N/A
rs2108622	*CYP4F2*	19	15851431	T	C	0.35	0.36	0.43^‡^	0.40	0.29	0.25	0.10	0.22	0.28

Freq: Frequency of Test Allele; Chr:Chromosome; MS: Metabolic Syndrome; N/A: Not Available; MAF: Minor AlleleFrequency. **GIH**: Gujarati Indians in Houston, Texas; **SAS**: South Asian; **NFE**: Non-Finnish European; **EAS**: East Asian; **AFR**: African/African American; **AMR**: Latino; **OTH**: Other.

*Refer to minor allele representing poor metabolism phenotype according to old nomenclature.

^**†**^Data obtained from 1000 Genome phase III.

^‡^Data obtained from HapMap.

**Table 2:** Comparison of Genotype Frequency Distribution of VIP Gene Variants in Punjabi Sikhs Healthy Controls (n = 752) vs. Global Populations from ExAC. Data are presented as p values.

Marker	Gene	South Asian		European	East Asian	African	Latino	Other
		GIH (n = 83)	SAS (n = 8256)	NFE (n = 33370)	EAS (n = 4327)	AFR (n = 5203)	AMR (n = 5789)	OTH (n = 454)
rs1801131	*MTHFR*	0.43	0.05	1.0E-05	1.0E-05	1.0E-05	1.0E-05	1.0E-05
rs1801133	*MTHFR*	0.38	1.0E-05	1.0E-05	1.0E-05	1.0E-05	1.0E-05	1.0E-05
rs1801159	*DPYD*	0.73	0.36	1.0E-05	1.0E-05	1.0E-05	1.0E-05	1.0E-05
rs1801265	*DPYD*	0.04	0.41	0.008	1.0E-05	1.0E-05	7.5E-04	0.17
rs4124874	*UGT1A1*	0.15^‡^	0.07	4.2E-06^‡^	1.1E-13^‡^	2.3E-14^‡^	0.11^‡^	N/A
rs887829	*UGT1A1*	0.84^‡^	0.30	8.9E-04^‡^	3.4E-15^‡^	9.3E-03^‡^	0.075^‡^	N/A
rs1045642	*ABCB1*	0.94	0.25	1.0E-05	1.0E-05	1.0E-05	1.0E-05	1.0E-05
rs2032583	*ABCB1*	0.52	0.41	6.7E-04	1.0E-05	5.6E-03	1.0E-05	7.4E-03
rs1799930	NAT2*6	0.38	0.39	1.0E-05	1.0E-05	1.0E-05	1.0E-05	1.0E-05
rs1799931	NAT2*7	0.63	4.1E-03	1.0E-05	1.0E-05	1.0E-05	1.0E-05	0.40
rs4244285	CYP2C19*2	0.82	0.08	1.0E-05	0.18	1.0E-05	1.0E-05	1.0E-05
rs4986893	CYP2C19*3	0.50	0.29	0.01	1.0E-05	0.01	0.01	0.04
rs1799853	CYP2C9*2	0.50	0.04	1.0E-05	1.0E-05	1.0E-05	0.08	4.7E-04
rs1057910	CYP2C9*3	0.28	0.37	1.0E-05	1.0E-05	1.0E-05	1.0E-05	1.0E-05
rs28371685	CYP2C9*11	0.27	0.27	0.19	0.10	0.000	0.41	0.18
rs1695	*GSTP1*	0.34	0.09	3E-05	1.0E-05	1.0E-05	1.0E-05	0.11
rs2306283	*SLCO1B1*	4.4E-04	1.0E-05	0.15	1.0E-05	1.0E-05	7E-03	1.0E-05
rs4149056	*SLCO1B1*	0.51	0.01	1.0E-05	1.0E-05	0.03	1.0E-05	1.0E-05
rs762551	CYP1A2*1F	0.04^‡^	0.12	5.3E-05^‡^	0.671^‡^	3.7E-02^‡^	5.5E-05^‡^	N/A
rs9923231	*VKORC1*	0.43^‡^	0.47	1.0E-05^‡^	1.0E-05^‡^	1.0E-05^‡^	1.0E-05^‡^	N/A
rs2108622	*CYP4F2*	0.15	6.8E-05	1.0E-05	1.0E-05	1.0E-05	1.0E-05	1.28E-04

Chr: Chromosome; MAF: minor allele frequency; P value for comparison of genotype frequencies were calculated either by Chi square or Fisher’s exact test; N/A: Not Available; **GIH**: Gujarati Indians in Houston, Texas; **SAS**: South Asian; **NFE**: Non-Finnish European; **EAS**: East Asian; **AFR**: African/African American; **AMR**: Latino; **OTH**: Other. ^**‡**^Data obtained from HapMap (European (CEU) n = 162; East Asian (CHB) n = 82; African (LWK) n = 83; Latino (AMR) n = 71).

**Table 3:** Details of pharmacogenomic actionable genes, associated drugs, and clinical effects.

Gene	Family/Name	Markers	Associated drug(s)	Clinical effect related to drugs
*MTHFR*	Methylenetetrahydrofolate reductase family	rs1801131, rs1801133	Methotrexate, Statins	Antineoplastic antimetabolite, immunosuppressant
*DPYD*	Dihydropyrimidine dehydrogenase	rs1801159, rs1801265	Fluorouracil, Tegafur	Antineoplastic antimetabolite
*UGT1A1*	UDP-glucuronosyltransferase family	rs4124874, rs887829	Belinostat, Irinotecan	Antineoplastic
*ABCB1*	ATP-binding cassette (ABC) transporters superfamily	rs1045642, rs2032583	Amitriptyline, Digoxin	Antidepressant
*NAT2*	N-acetyltransferase-2	rs1799930, rs1799931	Isoniazid, Sulfasalazine	Antibacterial agent used primarily as a tuberculostatic
*CYP2C19*	Cytochrome P450 superfamily	rs4986893, rs4244285	Clopidogrel, Omeprazole	Anticoagulant, anti-ulcer
*CYP2C9*	Cytochrome P450 superfamily	rs28371685, rs1057910, rs1799853	Warfarin	Anticoagulant
*GSTP1*	Glutathione S-transferase family	rs1695	Cisplatin, Fluorouracil	Antineoplastic
*SLCO1B1*	Solute carrier family	rs2306283, rs4149056	Irinotecan, Simvastatin	Anti-cancer, and Cholesterol lowering
*CYP1A2*	Cytochrome P450 superfamily	rs762551	Caffeine, Olanzapine, Leflunomide, Propafenone	Rheumatoid arthritis, Caffeine metabolism and myocardial infarction
*VKORC1*	Vitamin K-epoxide reductase complex	rs9923231	Warfarin	Anticoagulant
*CYP4F2*	Cytochrome P450 superfamily	rs2108622	Warfarin	Anticoagulant

Genotype frequencies of several VIP variants differed significantly in Sikhs compared to other major global populations as presented in Table 2. However, the allelic distribution in most VIP variants did not differ significantly between Punjabi Sikhs and GIH with the exception of three variants (rs1801265, rs2306283 and rs762551) encoding *DPYD, SLCO1B1*, and *CYP1A2* genes respectively (p value < 0.05) (Table 2**)**. Additionally, there was a significant difference in allelic distribution of rs1801133, rs1799931, rs2306283, and rs2108622 encoding *MTHFR*, NAT2*7, *SLCO1B1*, and *CYP4F2*, respectively, between Sikhs and SAS, and the difference remained significant even after the Bonferroni correction (p < 0.0018) (Table 2**)**. Moreover, the frequencies of low activity variants located in *MTHFR* gene (rs1801131 and rs1801133), were highest in Sikhs when compared with all global populations. Similarly, the frequency of a low activity *CYP4F2* variant (rs2108622), was also significantly higher in South Asian populations (p < 0.05) compared to all the other ethnic populations (Table 2**)**.

Interestingly, the PM phenotype encoded by CYP2C19*3 (rs4986893) was observed in Asian Indian and East Asian populations (SDS, GIH, and SAS (MAF: 0.004–0.005), EAS (0.07)), and was not seen in other European and Hispanic populations. (Table 1). On the other hand, the frequency of PM variant encoded by CYP2C9*11 (rs28371685) was observed in all Asian Indians, Sikhs (0.001), GIH (0.006), SAS (0.002), and NFE (0.002). The highest frequency for this variant was observed in African (AFR: 0.021), while this variant was monomorphic in the East Asian population **(**Table 1, Supplementary Table 2).

**Figure 1:**
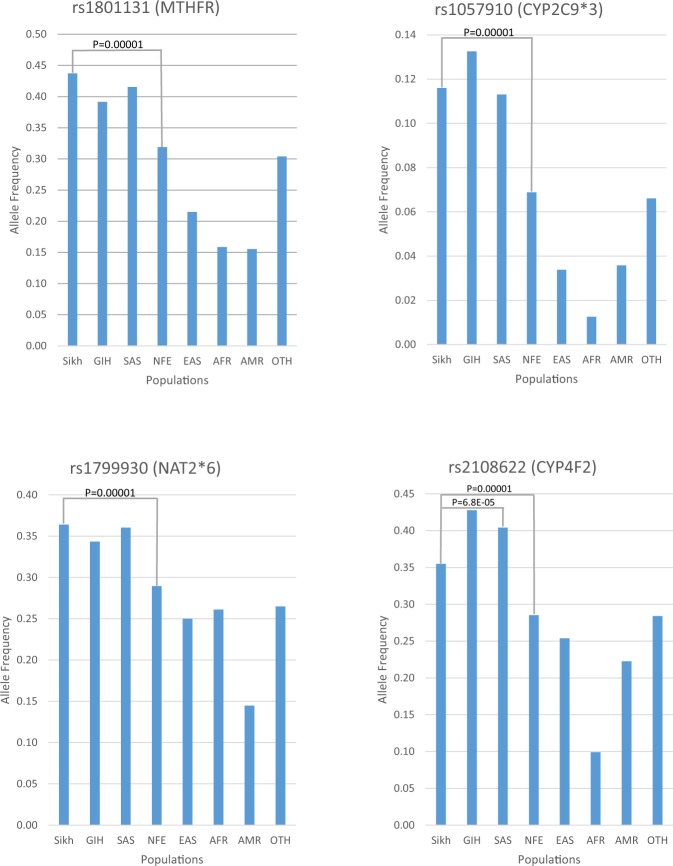
Allele frequency patterns of selected pharmacogenomic actionable genes in global populations. Data shows significant differences in the allele frequency of PM phenotype in Sikhs vs. South Asians, and Europeans. Sikh: The Punjabi Sikh populations from Northern India, (current study); GIH: Gujarati Indians in Houston, Texas (HapMap); SAS: South Asians; NFE: Non-Finnish European; EAS: East Asian; AFR: African/African American; AMR: Latino; OTH: Other. Source of data was from Exome Aggregation Consortium for all ethnic groups except GIH, which was obtained from 1000 Genomes.

Asian Indians and SAS had a significantly higher frequency of CYP2C9*3 (rs1057910 C) alleles (SDS: 0.12, GIH: 0.13, SAS 0.11) as compared to 0.07 in Europeans (p_genotype_ = 1.0E-05). While the frequency of ‘T’ risk allele at rs1801133 of *MTHFR* (C677T) gene was lower in Indians compared to Europeans (SDS: 0.20, GIH: 0.16, SAS 0.14 vs. NFE: 0.35), the ‘C’ allele of rs1801131 of *MTHFR* (E429A) was strikingly higher in SDS (0.44) compared to GIH (0.39), SAS (0.42) and NFE (0.32) (SDS vs. NFE p_genotype_ = 1.0E-05) (Tables 1,2). Additionally, the frequency of ‘C’ risk allele in rs4149056 (V174A) of the *SLCO1B1* gene was lower in Indians compared to Europeans (SDS: 0.04, GIH: 0.02, SAS: 0.05, and NFE: 0.16; SDS vs NFE p_genotype_ <1.0E-05). On the other hand, the frequency of variant corresponding to the NAT2*6 (rs1799930) allelic group was highest in Asian Indians (Sikhs (0.36) and GIH (0.34) and SAS (0.36) than in Europeans (0.29) and other populations see (Tables 1,2). Likewise, allele frequency *CYP4F2* (rs2108622) was highest in populations of Indian diaspora compared to other global populations. Allele frequency patterns of selected pharmacogenomics actionable genes [CYP2C9*3 (rs1057910), *CYP4F2* (rs2108622), *MTHFR* (rs1801131), and NAT2*6 (rs1799930)] in global populations are presented in Fig. 1. Taken together, highly diverse patterns for allele and genotype frequencies were observed in VIP variants among the Sikhs and other global populations.

## Discussion

After the completion of the human genome project, the list of gene polymorphisms influencing drug efficacy and risk for ADR events has steadily grown. It is well established that the differences in drug response across ethnic groups is due to diversity in drug target genes^24^. However, despite technological advancements, clinical implications of pharmacogenetics tests are still limited to a handful of (~20) gene-drug pairs called pharmacogenomics actionable or ADME genes. These gene tests have been partly adopted as the standard of care only in the United States and some other parts of the developed world. Surprisingly, the variant information in several culturally and ethnically diverse populations is not even available in a large part of the world.

South Asians, people with ancestors from the Indian subcontinent (e.g., India, Pakistan, Bangladesh and Sri Lanka), have a higher prevalence of cardiovascular disease (CVD) and a greater risk of CVD-associated mortality than European populations^25,26^. They also constitute 1/5^th^ of the entire globe. Moreover, because of the caste system, language, consanguinity, and distinct cultural and religious practices, populations of Indian diaspora constitute several small homogenous communities that display a great deal of genetic diversity^11,27,28^. Given that South Asians carry a disproportionally large burden of cardio-metabolic diseases, clinical implementation of preemptive screening of crucially important drug metabolizing genes would improve drug efficacy, and significantly reduce complications due to ADR events.

The present study examines the distribution of genetic variants in 21 VIP variants from 12 pharmacogenomic actionable genes in a Punjabi population of Sikhs from north India. Most of these genes are directly connected to the metabolism of commonly prescribed high-risk pharmaceuticals. Even though, the distribution of many VIP variants varied significantly across ethnic groups, the differences in frequencies of risk variants in 11 of the 12 genes was of significant interest in the Punjabi and GIH populations. More specifically, the low activity *MTHFR* (rs1801131) *allele* was significantly altered in Sikhs, Gujarati (GIH), and SAS compared to all other global populations. This risk allele with low enzyme activity (rs1801131) indeed had the highest frequency in Sikhs compared to all other ethnic groups.

The *MTHFR* gene is localized on chromosome 1p36.22, it encodes methylenetetrahydrofolate reductase that catalyzes the conversion of 5,10-methylenetetrahydrofolate to 5-methyltetrahydrofolate, a co-substrate for homocysteine remethylation to methionine. Deficiency of the MTHFR enzyme leads to hyper-homocysteinuria and thrombophilia. Individuals homozygous for T allele of C633T (rs1801133) or C allele of A1298C variant (rs1801131) have ~5–60% reduction in MTHFR activity^29,30^. Severely reduced enzyme activity is linked with developmental delays, seizures, schizophrenia disturbances, and ADHD^31^. *MTHFR* SNPs are also associated with acute cerebral stroke in young adults, premature CVD, and advanced arterial stenosis. Additionally, SNPs in *MTHFR* also affect the metabolism of statin drugs (atorvastatin)^32^, 5-fluorouracil, and methotrexate-based cancer and anti-inflammatory chemotherapy^33^.

The carriers of CYP2C19*2 and CYP2C19*3 alleles would be poor metabolizers of commonly used agents including clopidogrel (plavix) (anticoagulant), omeprazole (anti-ulcer), proguanil (antimalarial), and amitriptyline and nortriptyline (tricyclic antidepressants)^6,34,35^.

Clopidogrel is commonly prescribed for anti-platelet therapy to prevent ischemic events in patients with acute coronary syndrome, myocardial infarction, or following the placement of a coronary artery stent. Earlier studies suggest that about 1/3^rd^ of the patients taking clopidogrel do not respond effectively^36^ due to genetic variation in target genes. Clopidogrel is a prodrug that is activated by cytochrome P450-CYP2C19 enzyme into an active metabolite that ultimately inhibits platelet aggregation by binding to the P2Y12 receptor. The frequencies of the loss-of-function CYP2C19*2 (rs4244285) were significantly higher in Sikhs and South Asians (0.32 [Sikh], 0.33 [GIH], [0.34] SAS, and East Asians [0.31]). Similarly, the reduced function CYP2C19*3 (rs4986893) variant was only found in Sikhs, GIH and SAS, and EAS and not in other ethnic groups, showing a significantly higher prevalence in East Asians (0.07). A similar high prevalence of CYP2C19*2 (0.37) and CYP2C19*3 (0.022) phenotypes was also reported in a Tamilian population from South India^37^. Based on findings of TRITON-TIMI 38 trial^6^, the patients who were carriers of the reduced function CYP2C19*3 alleles had a higher rate of major adverse CV events than non-carriers when treated with clopidogrel. Such patients should be treated with prasugrel (theinopyridine), an FDA approved agent that does not undergo hepatic activation^38^.

Warfarin (Coumadin) is an anticoagulant commonly used to treat blood clots, deep vein thrombosis, and to prevent stroke in patients with arterial fibrillation. It prevents clot formation by blocking vitamin K epoxide reductase complex 1 (*VKORC1*). CYP2C9*2 and CYP2C9*3 are two major variants associated with reduced enzyme function that subsequently impact drug clearance compared to the wild type alleles. These patients carrying low activity alleles are at higher risk of bleeding and require a lower maintenance dose of warfarin^3^. Importantly, all South Asians including Punjabi (Sikh), GIH, and SAS have a higher frequency of the poor metabolizing CYP2C9*3 allele compared to Europeans. Incidentally, in ExAC data, all other populations have very low frequency of the CYP2C9*3 allele. Additionally, polymorphism (Val433Met) in the *CYP4F2* gene, represented by rs2108622, influences the warfarin dose due to reduced capacity of the Met (T) allele to metabolize vitamin K. Patient carriers of the Met allele require a higher warfarin dose to elicit the same anticoagulation response as the wild type carriers^4^. The frequency of Met allele carriers was significantly higher in GIH (0.43), Sikhs (0.36) and SAS (0.40) compared to 0.29 (p = 1.0E05) in Europeans (Fig. 1, Table 1). Further, genetic variation in the *VKORC1* gene also predicts warfarin dosing and response^39^. A low activity T allele of rs9923231 in the *VKORC1* is very common in East Asians (0.95; EAS) compared to Europeans (0.40; NFE) and South Asians [0.20 (Sikhs and SAS) and 0.19 (GIH)], and was lowest (0.06) in African (YRI). Because of ancestry differences, East Asian populations require a very low dose compared to African populations needing a very high dose of warfarin.

The influence of *CYP1A2* polymorphisms has been extensively studied in the metabolism of procarcinogens and subsequent susceptibility to various cancers^5,40^. Homozygous carriers of wild type ‘A’ alleles of rs762551 have been reported to rapidly metabolize caffeine, while, the ‘C’ allele (also known as CYP1A2*1F) carriers are slow metabolizers of caffeine. One investigation has reported the interaction of the CYP1A2*1F allele with impaired caffeine metabolism and the increased risk of myocardial infarction^41^. In a separate study, rheumatoid arthritis patients who were homozygous carriers of CYP1A2*1F (CC) alleles had over a 9 fold increase in toxicity with leflunomide compared to wild type allele ‘A’ carriers^42^. Notably, the allele frequency of slow metabolizer allele (CYP1A2*1F/‘C’) was significantly higher (p = 5.3E-05) in Sikhs (0.42) and GIH (0.51) compared to Europeans (0.29) (Fig. 1). Arguably, some earlier studies used restriction fragment length polymorphisms (RFLP) (rs762551A > C), and for some reason, have named the low frequency ‘C’ allele as a wild type allele which has resulted in conflicting reports on the effect of the ‘C’ allele on olanzapine metabolism^43^.

Isoniazid compounds are commonly used as anti-tuberculosis agents. Slow acetylation of isoniazid by *N*-acetyltransferase 2 (*NAT2*) has been reported as a rare event in some patients treated with isoniazid, which leads to the development of peripheral neuritis^44^. Earlier studies have reported that 10% of East Asians and 50% of Europeans exhibited a slow acetylator phenotype^45^. However, in this study, compared to all other global populations, South Asians (Sikhs (0.36), GIH (0.34), and SAS (0.36) had the highest frequency of *NAT2* ‘A’ (NAT2*6) allele of slow acetylation (Table 1). High frequency of NAT2*6 has been seen in the HapMap data of African populations from Kenya MKK (0.30) and LWK (0.28), compared to Europeans (0.27) and East Asians (0.20–0.25). Fundamentally, Africa and the Indian sub-continent are two of the most infested regions with tuberculosis; preemptive screening of *NAT2* alleles would be greatly helpful to prevent ADR events.

Although, the distribution of many of the VIP variants within the Indian populations (Sikhs and GIH) was nearly identical, the frequency of some of the PM phenotypes differed significantly. For instance, there was a significant difference in the distribution of PM alleles for *CYP4F2* rs2108622 [(0.36) Sikhs and (0.43) GIH]; *SLCO1B1* rs2306283 [(0.40) Sikhs and (0.55) GIH]; CYP1A2*1F rs762551 [(0.42) Sikhs and (0.51) GIH]; and *MTHFR* rs1801131 [(0.44) Sikhs and (0.39) GIH]. Thus, genetic screening of diverse ethnic groups from India will provide widespread comparison on the genetic spectrum of these important ADMEs. Indeed, recent genetic studies have reported a great deal of heterogeneity within Caucasian populations from Europe. For instance, in spite of the belief that Italy was a part of western European ethnicity, studies using genome-wide SNP data have shown a population structure on a fine–spatial scale in Italy, which is strongly influenced by geographical distance^46^. Similarly, studies on Finland and Icelandic populations have shown remarkable distinction between Finnish and western European populations, there was even substructure within the Finnish (founder) population^47^. These results further necessitate the need for future screening in ethnically diverse Indian and other South Asian and global populations.

In summary, our study has comprehensively examined the distribution of polymorphisms in 12 crucially important pharmacogenomic actionable genes in a large population dataset of Punjabi Sikhs and compared their similarities and differences to another south-western Indian population of Gujarati’s (GIH) from HapMap and Pakistani populations from ExAC. These data suggest potential benefits of optimizing genotype-guided therapy for commonly prescribed agents to reduce ADRs. Given the large-scale burden of CVD in India, we believe that our study is timely and provides valuable information on an understudied but important population of India. It also underscores the need for evaluation of other endogamous ethnic groups of India.

One limitation in our study is the lack of information on other Punjabi communities mainly Hindus and Punjabi Muslims and other sub-ethnic communities; their inclusion would have been more informative for such studies. Future multiethnic genetic surveys in entire Asian Indian and South Asian populations would be required to create foundations for clinically meaningful data banks. As this study was a retrospective evaluation in some compelling ADME genes linked to drug metabolism of commonly used drugs in CVD, cancers, and TB, it does not provide insight on the mechanism associated with the PM phenotype. Also, this study is limited to 12 highly important ADME genes and expansion of such evaluations in other ADME genes influencing metabolism of hundreds of other drugs will be required in future studies. Despite limitations, the genetic information on the prevalence of PM phenotypes of e.g., Warfarin, Clopidogrel, Statins, Methotrexate, etc. on Sikhs and South Asians would be of great interest to clinicians and pharmaceutical industry, as it 1) adds/complements to the existing pharmacogenomics data on global populations; 2) provides insights on the drug efficacy and toxicity; and 3) helps to identify high risk individuals within the Indian sub-ethnic groups.

Future advancements in sequencing technologies and its increased affordability would further enhance our understanding of genetic diversity and its implications in stratifying rapid, normal, and poor metabolizers before considering therapeutic options. These efforts will help in the design of population-optimized strategies that would account for differences in therapeutic response and reduce the incidence of ADRs and deaths due to treatment failures.

## Electronic supplementary material

Supplementary Information
