# Supplementary material for: Frequencies of poor metabolizer alleles of 12 pharmacogenomic actionable genes in Punjabi Sikhs of Indian Origin
Source: Sci Rep. 2018 Oct 24;8:15742. doi: 10.1038/s41598-018-33981-z (PMC6200732; doi:10.1038/s41598-018-33981-z)
Supplement: Supplementary file 1 — Supplementary Information [file 41598_2018_33981_MOESM1_ESM.pdf]

Frequencies of poor metabolizer alleles of 12 pharmacogenomic actionable genes in Punjabi Sikhs of Indian Origin

Dharambir K Sanghera

Department of Pediatrics, College of Medicine, University of Oklahoma Health Sciences Center, Oklahoma City, Oklahoma, USA

Department of Pharmaceutical Sciences, University of Oklahoma Health Sciences Center, Oklahoma City, OK, USA

Oklahoma Center for Neuroscience, University of Oklahoma Health Sciences Center, Oklahoma City, OK, USA

Harold Hamm Diabetes Center, University of Oklahoma Health Sciences Center, Oklahoma City, OK, USA

Cynthia Bejar

Department of Pediatrics, College of Medicine, University of Oklahoma Health Sciences Center, Oklahoma City, Oklahoma, USA

Bishwa Sapkota

Department of Pediatrics, College of Medicine, University of Oklahoma Health Sciences Center, Oklahoma City, Oklahoma, USA

Harold Hamm Diabetes Center, University of Oklahoma Health Sciences Center, Oklahoma City, OK, USA

Gurpreet S Wander

Hero DMC Heart Institute, Ludhiana, India

Sarju Ralhan

Hero DMC Heart Institute, Ludhiana, India

Supplementary Table 1: Clinical characteristics of study population stratified by metabolic syndrome (MS)

Traits	MS controls (n=752) Mean (SD)	MS cases (n=864) Mean (SD)
Age (years)	51.3 (14.3)	54.3 (11.1)
Gender (%males)	38.8	53.7
Waist (cm)	88.5 (10.9)	97.0 (10.9)
WHR	0.9 (0.1)	1.0 (0.1)
BMI (kg/m^2)	24.7 (4.3)	27.9 (4.9)
Systolic BP (mmHg)	130.6 (23.7)	145.4 (22.3)
Diastolic BP (mmHg)	78.8 (11.6)	85.8 (13.9)
FBG (mg/dL)	103.5 (30.9)	140.1 (55.1)
Cholesterol (mg/dL)	168.3 (50.8)	177.9 (51.5)
TG (mg/dL)	118.2 (60.2)	181.1 (90.1)
HDL (mg/dL)	39.3 (16.4)	33.5 (12.4)
LDL (mg/dL)	99.5 (35.8)	103.4 (41.8)

WHR - waist to hip ratio, BMI - body mass index, BP - blood pressure
FBG - fasting blood glucose, TG - Triglyceride, HDL - high density lipoprotein cholesterol
LDL - low density lipoprotein cholesterol, values are mean (SD)

Supplementary Table 2: Allele frequency distribution of rare PM variants in 1000 genome phase III data

Population	CYP2C19*3 (rs4986893)	CYP2C9*11 (rs28371685)
African (AFR)	0.002	0.024
American (AMR)	-	0.001
East Asian (EAS)	0.056	-
European (EUR)	-	0.002
South Asian (SAS)	0.012	0.001
Punjabi Sikh (SDS)	0.005	0.001
**Global**	**0.014**	**0.007**
